# Unique Roles of Phosphorus in Endochondral Bone Formation and Osteocyte Maturation

**DOI:** 10.1002/jbmr.294

**Published:** 2010-11-18

**Authors:** Rong Zhang, Yongbo Lu, Ling Ye, Baozhi Yuan, Shibin Yu, Chunlin Qin, Yixia Xie, Tian Gao, Marc K Drezner, Lynda F Bonewald, Jian Q Feng

**Affiliations:** 1 Department of Operative Dentistry and Endodontics, School of Stomatology, Fourth Military Medical UniversityXi'an, People's Republic of China; 2 Biomedical Sciences, Baylor College of Dentistry, Texas A&M Health Science CenterDallas, TX, USA; 3 Oral Biology, University of Missouri–Kansas CityKansas City, MO, USA; 4 Department of Medicine, University of Wisconsin and GRECC, VA Medical CenterMadison, WI, USA

**Keywords:** DMP-1, HYPOPHOSPHATEMIC RICKETS, FGF-23, PHOSPHATE HOMEOSTASIS, OSTEOCYTE

## Abstract

The mechanisms by which inorganic phosphate (P_i_) homeostasis controls bone biology are poorly understood. Here we used *Dmp1* null mice, a hypophosphatemic rickets/osteomalacia model, combined with a metatarsal organ culture and an application of neutralizing fibroblast growth factor 23 (FGF-23) antibodies to gain insight into the roles of P_i_ in bone biology. We showed (1) that abnormal bone remodeling in *Dmp1* null mice is due to reduced osteoclast number, which is secondary to a reduced ratio of RANKL/OPG expressed by osteoclast supporting cells and (2) that osteoblast extracellular matrix mineralization, growth plate maturation, secondary ossification center formation, and osteoblast differentiation are phosphate-dependent. Finally, a working hypothesis is proposed to explain how phosphate and DMP1 control osteocyte maturation. © 2011 American Society for Bone and Mineral Research.

## Introduction

Recently, we and other groups have demonstrated that mutations in *DMP1* result in autosomal recessive hypophosphatemic rickets (ARHR) in humans. This is characterized by rickets and the presence of large amounts of osteoid in bone (osteomalacia) and is accompanied by elevated circulating fibroblast growth factor (FGF-23).(1–4) Osteocytes in *Dmp1* null mice, the model used for human ARHR, regardless of whether they are newly formed or deeply embedded, continue to express many molecular markers of osteoblasts and osteoid osteocytes, such as alkaline phosphatase, type 1 collagen, and E11/gp38.(1) They also express elevated levels of FGF-23.(1) These observations suggest that DMP1, a protein highly expressed in osteocytes, might regulate the maturation of osteoid osteocytes directly or indirectly through FGF-23 regulation of phosphate homeostasis.(5)

Osteocytes, which are terminally differentiated osteoblasts, reside within the mineralized bone matrix and make up more than 90% to 95% of all bone cells in the adult skeleton. The differentiation of osteoblasts into osteocytes has been classified into several stages based on cell morphology and relative position in bone. These stages include osteoblasts residing on the bone surface, osteoblastic osteocytes or preosteocytes, osteoid osteocytes, and mature osteocytes embedded in a mineralized matrix.(6,7) As osteoblasts differentiate into mature osteocytes, they gradually reduce their cytoplasmic volume, protein synthesis, and secretion.(6) However, the molecular and cellular mechanism(s) governing this osteoblast differentiation process are largely unknown.

Classically, phosphate homeostasis has been viewed as being controlled by parathyroid hormone/1,25-dihydroxyvitamin D regulation of phosphate absorption in the intestine and reabsorption in the kidney.(8) However, recent findings suggest that FGF-23 is a potent phosphaturic hormone expressed predominantly by osteocytes in bone(1,9–10) that targets the kidney to promote renal excretion of phosphate.(11,12) These observations imply that bone functions as an endocrine organ, forming the bone-kidney axis in maintaining phosphate homeostasis.(1,13) In addition to *DMP1*, *PHEX* (a phosphate-regulating gene with homologies to endopeptidases on the X chromosome) also regulates FGF-23 expression in bone.(14) *PHEX* is expressed predominantly in osteoblasts and osteocytes.(15) *PHEX* mutations in mice and humans result in autosomal dominant hypophosphatemic rickets, accompanied by elevated circulating FGF-23, a phenotype identical to that of *Dmp1* null mice.(1,9) These observations suggest that elevated circulating FGF-23 levels and hypophosphatemia are the pathogenic factors involved in both *Phex* and *Dmp1* mutant mice and that presence of hypophosphatemia and FGF-23 may inhibit osteoblast to osteocyte differentiation. Note that FGF-23 also plays a role in skeletal mineralization and chondrocyte differentiation that is independent of phosphate homeostasis.(16)

Based on observations that *Dmp1* null mice show osteomalacia accompanied by hypophosphatemia and elevated FGF-23 levels, this study set out to further characterize the skeletal abnormalities in *Dmp1* null mice and determine the mechanisms responsible for those defects. We first determined whether *Dmp1* null mice show abnormalities in bone remodeling and osteoclast function. Next, mechanistic experiments were performed to determine whether restoration of phosphate or blocking the activity of serum FGF-23 can rescue the skeletal abnormalities in the *Dmp1* null mice. These studies have highlighted key roles for FGF-23 and phosphate in mediating the DMP1 phenotype.

## Materials and Methods

### Mice

*Dmp1* knockout (KO) mice with targeted deletion of exon 6 have been described previously.(17) The mice in CD-1 background were fed with autoclaved Purina rodent chow (5010; Ralston Purina, St. Louis, MO, USA) containing calcium, 0.67% phosphorus, and 4.4 IU of vitamin D per gram. The age-matched wild-type or heterozygous mice were used as control because there is no an apparent difference between the wild-type and the heterozygous mice.(1,18) All animal protocols were approved by the Institutional Animal Care and Use Committee.

### Injections of anti-FGF-23 neutralizing antibodies

Peritoneal injections of *monoclonal* FGF-23 antibodies [FN1 for against the N-terminal and FC1 for against the C-terminal fragments (see Yamazaki and colleagues for details(19,20)) or PBS into *Dmp1* KO or the age-matched control mice (4 to 6 mice/group) started 6 days after birth every other day at dosages between 25 and 40 µg per pup based on the age. Mice were euthanized on days 15 and 28.

### Metatarsal organ culture

Metatarsal organ cultures were performed to investigate the effects of phosphate on the development of secondary ossification centers. Briefly, the three central metatarsal bone rudiments were dissected out from hind limbs of 8-day-old *Dmp1* null mice and the age-matched control mice. They were cultured in a 24-well plate, one bone per well, in 1 mL of α-modified minimum essential medium (α-MEM; Gibco BRL, Carlsbad, CA, USA) supplemented with 0.5% fetal calf serum (FCS), 0.05 mg/mL of ascorbic acid, 0.05 µg/mL of gentamicin, 100 units/mL of penicillin, 50 µg/mL of streptomycin, and various concentrations of β-glycerophosphate. The explants were cultured for 8 days with the medium changed every other day.

### Calvarial cell culture

Calvarial cells, enriched for cells with an osteoblast phenotype, were isolated from *Dmp1* null pups and age-matched control pups at the age of 5 days using procedures described by Feng and colleagues.(21) Briefly, first-passage cells were plated at a density of 1.5 × 10^4^ cells/well in 24-well tissue culture dishes (Costar, Corning, NY, USA). Cells were grown to confluence in α-MEM supplemented with 7% FCS. The medium then was changed to α-MEM supplemented with 7% FCS, 100 µg/mL of of ascorbic acid (Sigma, St Louis, MO, USA), and 5 mM of β-glycerophosphate (Sigma). This was defined as day 0. Subsequently, the medium was changed every 2 days until cell cultures were stopped at day 14 and stained using the alizarin red procedure.

### Osteoclast culture and pit-formation assay

Osteoclasts were generated by coculturing splenocytes from 6-week-old mice with either 1,25-dihydroxyvitamin D_3_ [1,25(OH)_2_D_3_] or in the presence of recombinant macrophage colony-stimulating factor (M-CSF) and receptor activator of NF-κB ligand (RANKL) supplements as described previously.(22,23) The highly enriched bone marrow mesenchymal cells also were used for RNA preparations for *Rankl* and *Opg*. In the experiments using the coculture assay, individual pairs of a *Dmp1* KO and control mice were used to initiate two separate in vitro cultures in 48-well plates and to compare the osteoclast differentiation and bone resorption between the cells derived from KO and control mice in a CD-1 background. The experiment was repeated four times. The M-CSF/RANKL supplement was used to induce osteoclast formation on tissue culture slides with 2-cm^2^ chambers containing 1 × 10^5^ splenic cells. All cultures were initiated in medium containing DMEM supplemented with 10% FCS, 20 ng/mL of human M-CSF (R&D Biosystems, Minneapolis, MN, USA), and 50 ng/mL of RANKL + 50 µg/mL of ascorbate + 100 nM dexamethasone. The cultures were processed for analysis on day 6. In addition, in the latter case, for each genotype, one plate was used to perform tartrate-resistant acid phosphatase (TRAP) staining to estimate the number of TRAP^+^ osteoclasts. With our protocol, TRAP^+^ cells accounted for approximately 95% of the total number of cells in both the wild-type and KO cultures.

### In vitro bone-resorption assay

The experimental procedure is very similar to that for the in vitro culture of the osteoclast experiments described earlier except for the addition of one dentin slice in each culture.(23) After 9 days of coculture, one set of slices was processed for TRAP staining. Another set of the slices was incubated for an additional 3 days and then fixed and stained with toluidine blue for photographs of excavated pits. The experiment was repeated three times.

### RT-PCR and real-time PCR

To determine the ratio of RANKL/OPG expressed by the osteoclast precursor supporting cells in *Dmp1* KO mice, total RNA was isolated from the midshaft of *Dmp1* control and KO femurs (4 for each) using the RNeasy Mini Kit (Qiagen, Inc., Valencia, CA, USA) and then reverse transcribed into cDNA using SuperScript III First-Strand Synthesis SuperMix for quantitative reverse-transcriptase polymerase chain reaction (qRT-PCR; Invitrogen, Inc., Carlsbad, CA, USA). Real-time PCR was carried out with the Light Cycler 480 Real-Time PCR System (Roche Molecular Biochemicals, Mannheim, Germany) using SybrGreen PCR Master Mix (Applied Biosystems, Foster City, CA, USA) with an initial denaturation at 95 °C for 10 minutes, followed by 45 cycles at 95 °C for 30 seconds, 60 °C for 1 minute, and 72 °C for 30 seconds. The PCR product accumulation was monitored by the increase in fluorescence intensity caused by binding of SybrGreen to double-stranded DNA. The primer sequences used are *RANKL*, forward: 5'-GAA ACT CAC AGC CCT CTC TCT TG-3', and reverse: 5'-GCA TCG GAA TAC CTC TCC CAA TC-3'; *OPG*, forward: 5'-TAC CTG GAG ATC GAA TTC TGC TT-3', and reverse: 5'-CCA TCT GGA CAT TTT TTG CAA A-3'), *GAPDH*, forward: 5'- ACC ACA GTC CAT GCC ATC AC -3', and reverse: 5'- TCC ACC ACC CTG TTG CTG TA-3'.

### Fluorochrome labeling of the mineralization front

To visualize bone mineralization in mice, double fluorescence labeling was performed as described previously.(24) Briefly, mice were first intraperitoneally injected with calcein green (5 mg/kg), followed by injection of an alizarin red label (5 mg/kg i.p.; Sigma-Aldrich) 5 days later. Mice were euthanized 48 hours after injection of the second label, and the bones were removed and fixed in 70% ethanol for 48 hours. The specimens were dehydrated through a graded series of ethanol (70% to 100%) and embedded in methyl methacrylate without prior decalcification. Then 15-µm sections were cut using a Leitz 1600 saw microtome (Ernst Leitz Wetzlar GmbH, Wetzlar, Germany). The unstained sections were viewed under epifluorescent illumination using a Nikon (Melville, NY, USA) E800 microscope interfaced with the Osteomeasure histomorphometry software (Version 4.1, Atlanta, GA, USA).

### Radiography, micro–computed tomography (µCT), and backscattered scanning electron microscope (SEM)

The long bones or vertebrae were dissected and X-rayed using a Faxitron MX-20 (Faxitron X-Ray, Lincolnshire, IL, USA) with digital image-capture capabilities. The long bones, vertebrae, and metatarsals were scanned in a µCT imaging system (µCT35; Scanco Medical, Bassersdorf, Switzerland). The nondecalcified tibias were scanned by a FEI/Philips XL30 field-emission environmental SEM (Hillsboro, OR, USA) using a method described previously.(18)

### Histology

For paraffin block preparations, specimens were fixed in freshly prepared 4% paraformaldehyde in PBS (pH 7.4), decalcified, and embedded in paraffin by standard histologic procedures, as described previously.(25) Then 5-µm sections were cut and dried. Sections were used for safranin-O staining, immunohistochemistry, toluidine blue staining (Sigma-Aldrich), TRAP staining, and in situ hybridization using digoxigenin-labeled antisense RNA (cRNA) probes, as described previously.(1) For immunohistochemistry, the following antibodies were used: DMP1 polyclonal antibody,(26) E11,(27) sclerostin polyclonal antibody (R&D Biosystems), and osterix monoclonal antibody (Abcam, Cambridge, MA, USA). For undecalcified bones, specimens were embedded in methyl methycrylate and cut at 6-µm thickness using a Leica 2165 rotary microtome (Ernst Leitz Wetzlar). Undecalcified sections were stained by von Kossa staining,(17) Goldner's masson trichrome staining,(1) alizarin red/alcian blue staining,(17) and alkaline phosphatase (ALP) cytochemistry.(28)

### Serum biochemistry

Serum calcium, phosphorus, and FGF-23 levels were determined as described previously.(1) Briefly, serum calcium concentration was analyzed using a colorimetric calcium kit (Stanbio Laboratory, Boerne, TX, USA). Serum phosphorus was measured by the phosphomolybdate/ascorbic acid method. Serum FGF-23 level was determined by a full-length FGF-23 ELISA kit (Kainos Laboratories, Tokyo, Japan). Data were analyzed using one-way ANOVA with Bonferroni correction.

### Statistical analysis

Data analysis was performed with the Student's *t* test for two-group comparison and with one-way ANOVA for multiple-group comparison. If significant differences were found with one-way ANOVA, the Bonferroni method was used to determine which groups were significantly different from others. The quantified results are represented as the ±SEM. *p* < .05 was considered statistically significant.

## Results

### Abnormal bone remodeling in *Dmp1* null mice is accompanied by altered osteoblast function and reduced osteoclast formation and function

**Fig. 1:**
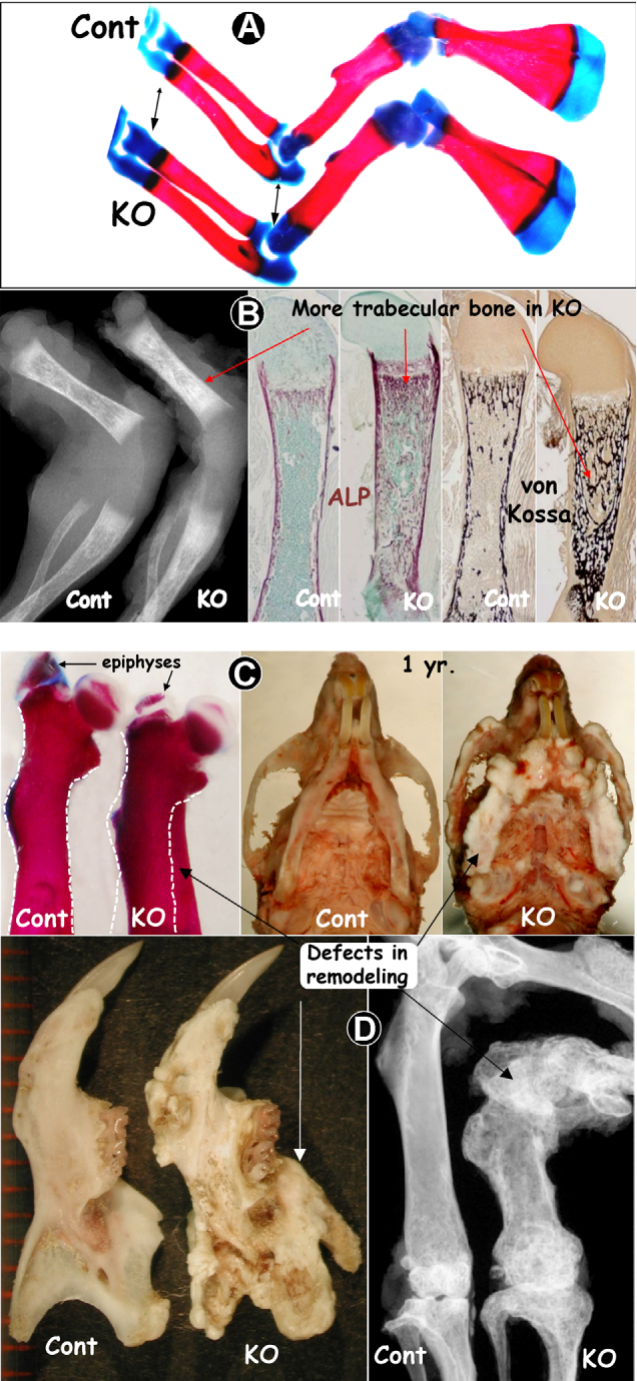
Abnormal bone remodeling in the *Dmp1* null mouse (*Dmp1* KO, *right panels*), which is exacerbated with age. (*A*) Photographs of 3-day-old forelimbs stained with alizarin red/alcian blue showing that the KO cartilage is remodeled less than control. (*B*) Radiographs of 3-day-old hind limbs of *Dmp1* control and KO images showing more trabecular bone in the KO bone marrow, which is further confirmed by ALP enzyme activity and von Kossa staining. (*C*, *D*) Photographs of alizarin red–stained femur (day 17), craniofacial bones (1 year), mandibular bone, and femur (1 year, X-ray) display severe defects of bone remodeling with age.

Although *Dmp1* null (*Dmp1* KO) newborns display no apparent skeletal abnormalities,(17) differences in both cartilage and bone become evident on day 3, as reflected by comparison of alizarin red/alcian blue–stained forelimbs (Fig. 1*A*). There is also a greater accumulation of trabecular bone in the bone marrow space as well as expanded metaphyses (Fig. 1*B*, *left panel*). This is further supported by increased alkaline phosphatase enzyme activity and the expanded von Kossa staining (Fig. 1*B*, *middle* and *right panels*) and abnormal morphology on day 17 (Fig. 1*C*, *left panel*). Our previous data(1) suggest that there may be a delay in osteoblast to osteocyte transition in *Dmp1* KO mice, which potentially would explain the increase in alkaline phosphatase activity. In the aged animal at 1 year, the development of large bony protuberances occurs in all bones examined (Fig. 1*C*, *middle* and *right panels*, *D* and Supplemental Fig. S1), suggesting that the abnormality of bone remodeling progresses with age.

**Fig. 2:**
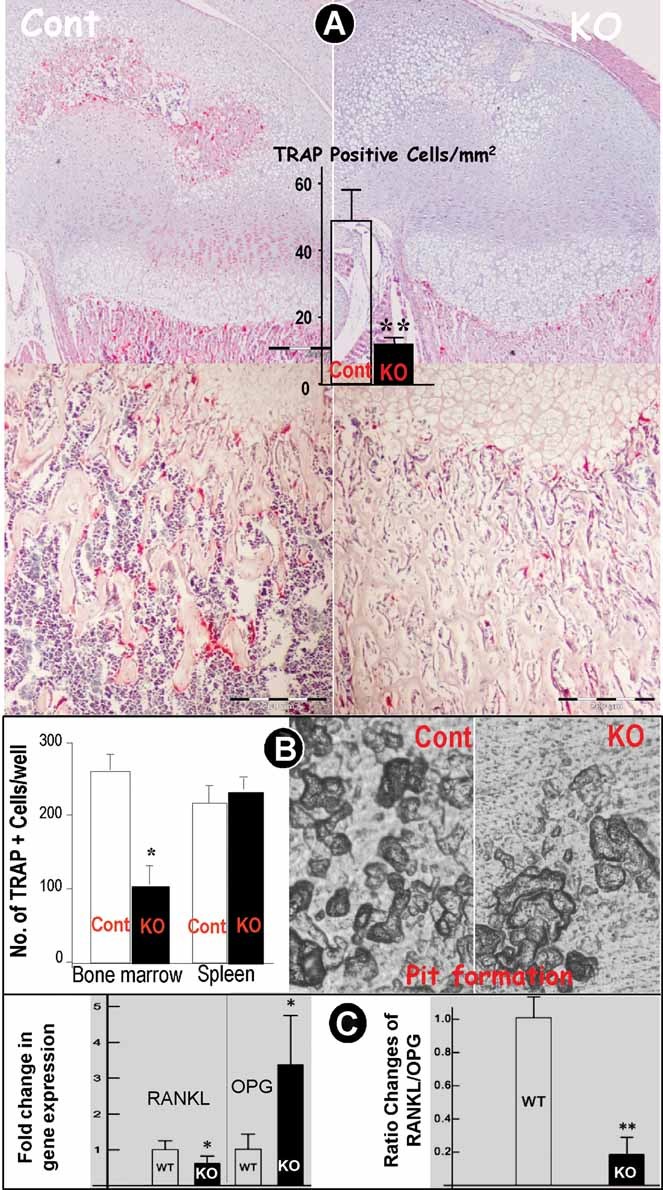
Reductions of osteoclasts in *Dmp1* null mice. (*A*) TRAP stained 3-week-old femurs showed a sharp reduction in osteoclast number (data are mean ± SEM from *n* = 4 to 6 animals). **p < .01 using Student's *t* test. (*B*) Bone marrow, a source of both osteoclast precursors and their supporting cells, from *Dmp1* KO mice only generated half the number of osteoclasts as that from control mice. However, when stimulated with RANKL and M-CSF, spleen, as a source of osteoclast precursors, from the *Dmp1* KO mice generated equivalent numbers of osteoclasts and pit formation on dentin as spleen from the control mice (data are mean ± SEM from *n* = 4 to 6 animals/3 replicates). *p < .05 using Student's *t* test. (*C*) Real-time PCR analysis showed that *Dmp1* KO long bones express less RANKL but three times more OPG, with a ratio of RANKL to OPG of 1:5 than in the control (data are mean ± SEM from 3 replicates). *p < .05; **p < .01, using Student's *t* test.

To understand the mechanisms underlying the abnormal bone remodeling, we examined osteoclast formation and function in *Dmp1* KO mice. The number of TRAP^+^ (a marker of osteoclast enzymes) cells was sharply reduced in *Dmp1* KO long bone in both the epiphyses and the metaphyses at 2 weeks (Fig. 2*A*). Consistent with this, the number of osteoclasts formed in bone marrow cultures from *Dmp1* KO mice in the presence of 1,25(OH)_2_D_3_ was less than 50% of that in control (Fig. 2*B*, *left bar*). However, *Dmp1* KO osteoclast precursors can differentiate normally when provided with the necessary factors RANKL and M-CSF because the number of osteoclasts formed by *Dmp1* KO spleen cells (osteoclast progenitors) is identical to that of control spleen cells (Fig. 2*B*, *right bar*). Yet resorption lacunae or pit formation on dentin, an assay for testing osteoclast function, was equivalent for both the *Dmp1* KO mice and the controls (Fig. 2*B*, *right panel*), suggesting that the *Dmp1 KO* precursors and mature osteoclasts are normal. Together, these data suggest that the abnormalities in remodeling in *Dmp1* KO mice are related to both the altered osteoblast function and the reduction of the number of osteoclasts. The osteoclast defect appears to reside in cells of the stromal/osteoblast lineage that support osteoclast formation rather than an intrinsic defect in the osteoclasts themselves (Fig. 2*B*). In search of the mechanism by which the number of *Dmp1 KO* osteoclasts is reduced, we performed both RT-PCR (Supplemental Fig. S2) and qPCR (Fig. 2*C*) analyses using long bone samples with the bone marrow preserved and showed that the *Dmp1* KO long bone expressed less RANKL and three times more OPG with a 1:5 ratio of RANKL to OPG than the control (Fig. 2*C*).

### Roles of phosphorus in the formation of secondary ossification centers

**Fig. 3:**
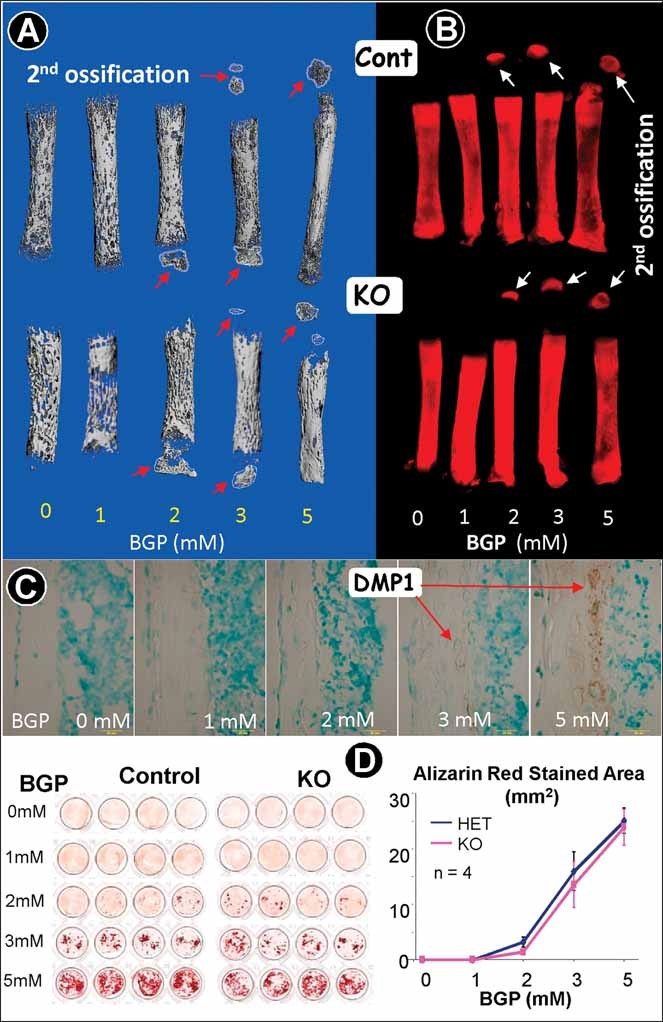
Ex vivo secondary ossification in metatarsals is phosphate dose dependent in both *Dmp1* control and KO osteoblasts. (*A*) Representative µCT images of 8-day-old mouse metatarsal organ cultures showing that ex vivo formation of both *Dmp1* control and KO epiphyses is (-glycerophosphate (BGP) dose dependent. (*B*) Representative alizarin red–stained images of 8-day-old mouse metatarsal organ cultures showing that the ex vivo formation of both *Dmp1* control and KO epiphyses is BGP dose dependent. (*C*) The DMP1 expression pattern appears to be BGP dose dependent in the cultured wild-type metatarsal. (*D*) Alizarin red–stained images of newborn mouse calvarial cultures showing that the mineralized nodule formation is BGP dose dependent in both *Dmp1* control and KO cultures.

The *Dmp1* KO phenotype is associated with hypophosphatemia and elevated FGF-23 concentrations. One or both of these may be critical mediators of the skeletal defects. In support of this hypothesis, we have shown previously that a high-phosphate diet significantly improves bone mineralization in the *Dmp1* KO tibia.(1) In addition, the high-phosphate diet also restored the defects in the growth plate of *Dmp1* KO mice.(1) Nevertheless, the high-phosphate diet only partially rescued the osteomalacia phenotype in *Dmp1* KO mice.(1) This is likely due to the fact that the *Dmp1* KO phenotype begins postnatally,(18) but the correction of phosphate homeostasis by the high-phosphate diet was started at week 3 after weaning.(1) To overcome this limitation and to avoid the potential influence of circulating factors, such as parathyroid hormone (PTH), 1,25(OH)_2_D_3_, and FGF-23, ex vivo experiments were performed in which the phosphate levels could be controlled experimentally using a metatarsal organ culture system. In this experiment, we showed a dose-dependent correlation between increasing phosphate and the formation of secondary epiphyseal ossification in 8-day-old metatarsals cultured for 8 days in both the control and *Dmp1* KO pups (Fig. 3*A*, µCT images; Fig. 3*B*, alizarin red stain, photographic images of metatarsals). Furthermore, we showed that the expression level of DMP1 in the wild-type metatarsal culture was positively correlated with the BGP level (Fig. 3*C*). Interestingly, we also demonstrated a dose-dependent correlation between the increasing phosphate and the formation of mineralized nodule in the calvarial cell isolated from both the control and the *Dmp1* KO pups (Fig. 3*D*). These data suggest that the delayed formation of secondary ossification centers is mainly due to the hypophosphatemia.

### Pathogenic roles of FGF-23 in *Dmp1*-KO mice

Having established that phosphate levels are key for manifestation of the *Dmp1* KO phenotype, we next determined the relative role of elevated FGF-23, a potent phosphaturic hormone that promotes renal phosphate excretion.(11,12,29) To test whether restoration of *Dmp1* KO phosphate homeostasis could rescue the *Dmp1* KO phenotype, we injected neutralizing antibodies against FGF-23(20) into *Dmp1* KO mice.

**Fig. 4:**
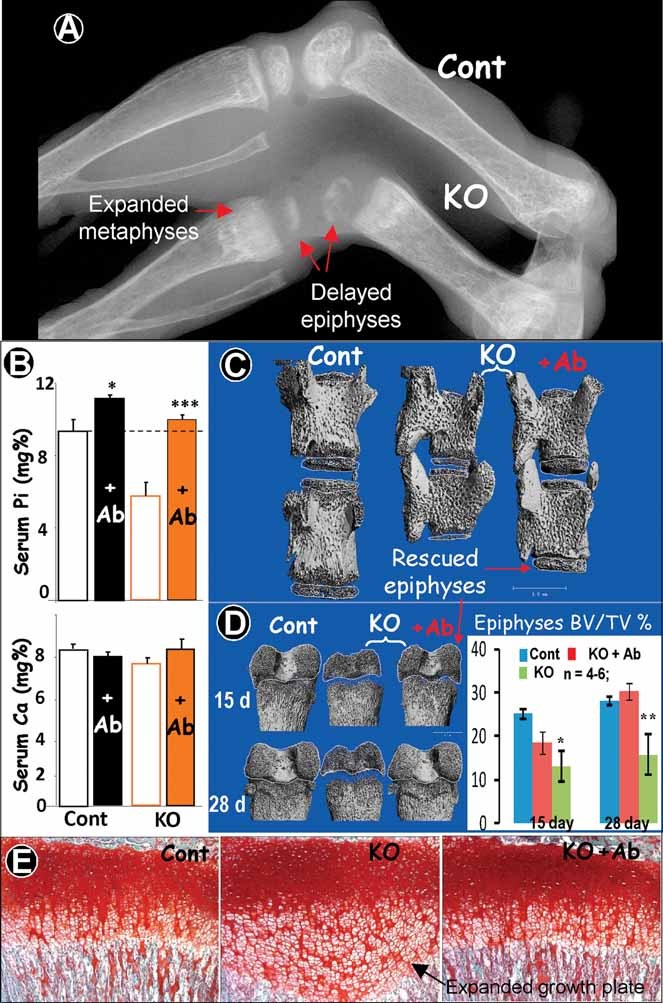
Rescue of *Dmp1* KO phosphate homeostasis and secondary ossification using anti-FGF-23 antibody injections. (*A*) Representative X-ray images of *Dmp1* KO long bones showed the delayed secondary ossification (epiphyses formation) and expanded metaphyses (day 14). (*B*) Injections of FGF-23 Abs in the control and KO mice for 9 days starting from day 6 significantly increased the serum P_i_ level in control mice and restored P_i_ levels to control levels in *Dmp1* KO mice with little effect on serum Ca levels (*lower panel*) (data are mean ± SEM from *n* = 4 to 6 animals). *p < .05; ***p < .01 compared with PBS using Student's *t* test. (*C*, *D*) µCT data showed partial (for 9-day injections) or full (for 22-day injections) rescue of epiphyses formation in the KO vertebrae (*C*) and femurs (*D*) (data are mean ± SEM from *n* = 4 to 6 animals). *p < .05; **p < .01 compared with control. (*E*) Safranin-O data showed full rescue of growth plate formation in the KO tibias.

The *Dmp1* KO mice showed delayed formation of secondary ossification centers, as demonstrated by radiography (Fig. 4*A*), which was used as a readout for testing the role of P_i_ in *Dmp1* KO mice. As expected, injections of FGF-23 antibodies restored serum phosphate levels (Fig. 4*B*). We then showed the rescue of secondary ossification defects in vertebrae (Fig. 4*C*) and femoral bone (Fig. 4*D*), as determined by µCT analysis. Quantitation confirmed that the BV/TV% in *Dmp1* KO mice was restored to the control level by 28 days after injections of FGF-23 antibodies. We also showed a full rescue of the growth plate (Fig. 4*E*), which is in agreement with the high-P_i_ diet rescue.(1)

**Fig. 5:**
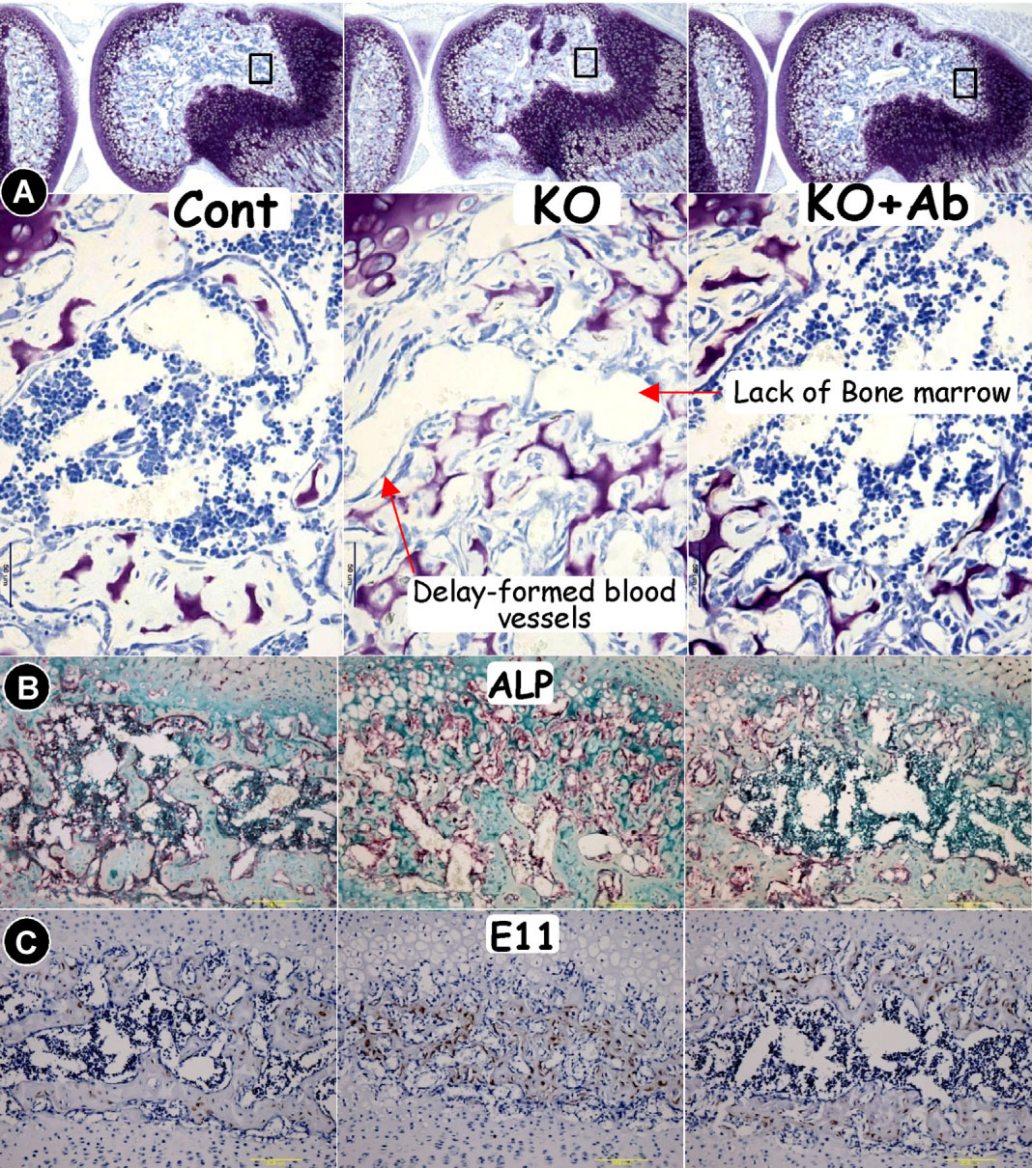
Rescue of the delay in marrow cavity formation in *Dmp1* KO mice by injections of FGF-23 antibodies (control, *left panels*; KO, *middle panels*; and KO + Ab injection, *right panels*) Injections of FGF-23 Abs accelerated bone marrow formation in the KO femur epiphyses, as documented by toluidine blue staining (*A*, showing a delay in formation of blood vessels and lack of bone marrow), ALP immunohistochemistry staining (*B*, signal in red, showing residual ALP stained trabecular bone in the center of the epiphyses and lack of formation of a marrow cavity), and E11 immunostain (*C*, signal in brown, showing the E11-stained trabecular bone in the center of the epiphyses and lack of formation of a marrow cavity).

Histologic analyses using toluidine blue staining (an assay revealing bone marrow cells and blood vessels; Fig. 5*A*), ALP (an early marker for bone formation; Fig. 5*B*), and E11 (a marker for newly formed osteocytes; Fig. 5*C*) showed that there was delayed blood vessel formation and bone marrow formation in *Dmp1* KO mice and that the injections of FGF-23 antibodies rescued these abnormalities.

**Fig. 6:**
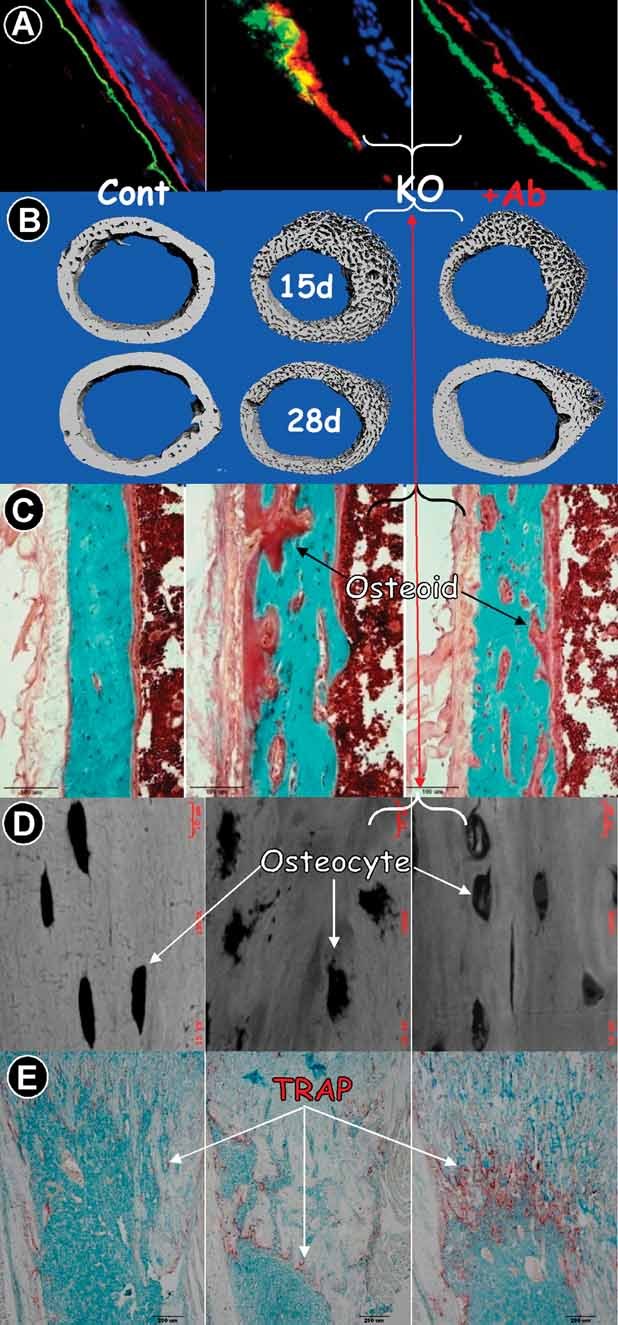
Partial rescue of the malformed long bone in *Dmp1* KO mice by injections of Anti-FGF-23 Ab (control, *left panels*; KO, *middle panels*; and KO + Ab injection, *right panels*). (*A*) Confocal microscope images of fluorochrome labeling counterstained with DAPI in 7-week-old mouse tibias (*red* = alizarin red; *green* = calcein; *blue* = DAPI). Note that the high-P_i_ diet restored the bone-formation labeling pattern in KO tibias to a pattern resembling control; however, the label is still thicker in width than the control. (*B*) Representative µCT images show improvement of cortical bone formation by injection of FGF-23 Ab at days 15 and 28 because the bone is less porous. (*C*) Goldner's staining shows a minor reduction in the amount of osteoid in KO cortical bone that received FGF-23 antibodies. (*D*) Backscattered SEM displayed a partial restoration of osteocyte morphology, although the mineralization was largely unchanged (the mineral content is shown in white). (*E*) Representative TRAP-stained femur sections showed that *Dmp1* null osteoclasts were distributed mainly on the metaphysis surface, and there were few trabeculae (*middle panel*). In contrast, there were more TRAP^+^ cells plus more trabeculae formed in the FGF-23-injected metaphysis, suggesting a partial rescue in osteoclastogenesis (*right panel*).

Treatment with FGF-23 antibodies also improved the abnormalities in cortical bone observed in *Dmp1* KO mice, as shown in Fig. 6. Note that with FGF-23 antibody treatments, the diffuse fluorochrome labeling pattern normally seen in the *Dmp1* KO mice (and other models of osteomalacia) is restored to a pattern resembling that in control mice, with discrete lines of calcein and alizarin red labeling, suggesting a more normal bone-formation rate (Fig. 6*A*). Bone quality, osteocyte morphology, and osteoclast number also were partially improved, as shown by less cortical porosity (Fig. 6*B*), reduced amounts of osteoid (Fig. 6*C*), improved osteocyte surface (Fig. 6*D*), and an increase in osteoclasts in the trabecular region (Fig. 6*E*).

**Fig. 7:**
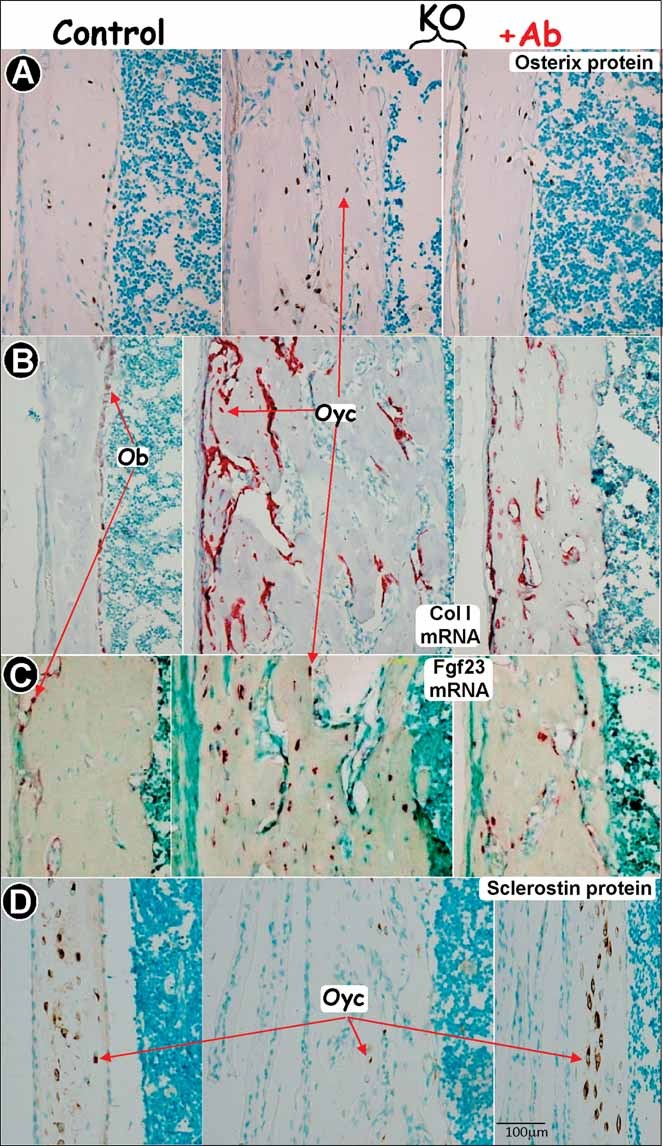
Rescue of molecular markers in the *Dmp1* KO osteoblast (Ob) and osteocyte (Oyc) by injections of FGF-23 antibodies (control, *left panels*; KO, *middle panels*; and KO + Ab injection, *right panels*). (*A*) Immunohistochemistry assay showed an increase in osterix expression in *Dmp1* null osteocytes and a return to the control level in the KO osteocytes injected with FGF-23 Ab. In situ hybridization shows a decrease in *Col1a1* mRNA (*B*, signal in red) and *Fgf23* mRNA (*C*, signal in red) in *Dmp1* KO osteoblasts and osteocytes injected with FGF-23 Ab. Immunohistochemical staining for sclerostin showed a reduction in osteocytes from *Dmp1* KO animals compared with control, which is restored in osteocytes from animals injected with FGF-23 Ab (*D*).

Histologic analyses showed that FGF-23 antibody treatments also partially reversed the increase in osterix, Col1a1, and FGF-23 expression normally seen in *Dmp1* KO mice (Fig. 7*A–C*) and restored expression of sclerostin, a marker of mature osteocytes that is largely undetectable in *Dmp1* KO osteocytes (Fig. 7*D*). These data suggest that FGF-23 antibodies can rescue the defect in osteocyte maturation in *Dmp1* KO mice. This is likely mediated through the effects of the neutralizing antibody to restore phosphate levels (Fig. 4*B*).

## Discussion

We previously showed that DMP1 and phosphate homeostasis are critical for bone and tooth formation using a *Dmp1* null animal model, that is, a hypophosphatemic rickets/osteomalacia model.(1,18,30) However, how phosphate homeostasis and DMP1 contribute to the mineralization remains unclear. In this study, we used the ex vivo culture of metatarsal organs, the primary calvarial cell culture, and application of neutralizing FGF-23 antibodies to investigate the specific roles of P_i_ and DMP1 in bone biology during postnatal development. Our key findings are (1) that *Dmp1* null mice develop defective bone remodeling owing to abnormal osteoblast formation and (2) that restoration of P_i_ homeostasis in *Dmp1* null mice via injections of anti-FGF-23 antibodies i.p. fully rescue defects in the growth plate and the secondary ossification process.

*Dmp1* null mice develop severe skeletal abnormalities with age, suggesting a defect in the bone-remodeling process, which normally is carried out by osteoblasts, which form bone, and osteoclasts, which resorb bone. Indeed, our previous study(1) and the current study have shown that the *Dmp1* null osteoblasts have altered functions. They continuously express many osteoblast-specific genes, such as osterix, Col1a1, osteocalcin, FGF-23, and E11, and fail to differentiate into mature osteocytes. This defect in osteoblast differentiation apparently is caused by hypophosphatemia because the isolated calvarial osteoblasts mineralize normally ex vivo if sufficient phosphate is provided (Fig. 3*D*). Furthermore, we have shown that the *Dmp1* null bone displays a reduced number of osteoclasts. This reduction in osteoclast formation is secondary to the defects in cells of the stromal/osteoblast lineage because these null cells express a reduced ratio of RANK/OPG in *Dmp1* null bone. This pathologic change could be caused by both the intrinsic osteoblast defect owing to a loss of DMP1 and an indirect role of hypophosphatemia on *Dmp1* null osteoblasts because restoration of P_i_ by injections of Fgf23Ab greatly improves osteoclast numbers and bone remodeling (Fig. 6*E*). However, we cannot rule out the possibility that hypophosphatemia also may adversely affect osteoclast formation and function directly in vivo.

PTH normally plays an important role in the regulation of osteoclastogenesis: A continuous high level of PTH stimulates this process, whereas a sustained low level inhibits osteoclast formation.(29) The best example is primary hyperparathyroidism, where a high level of PTH leads to an increase in osteoclast activity and bone loss. Interestingly, the PTH level in the *Dmp1* null mouse is constantly higher (5 to 10 times) than that in age-matched control animals,(1) but the osteoclast number is less than 50% of that in controls (Fig. 2). Apparently, the defect in osteoclastogenesis observed in *Dmp1* null mice cannot be explained by the physiologic catabolic role of PTH. On the other hand, defects in osteoclastogenesis will lead to a massive trabecular bone accumulation in the entire bone marrow plus a lack of tooth eruption. But none of these changes is observed in this animal model except for an expanded metaphysis. Does this mean that the number of osteoclasts would be even lower if the PTH factor were removed from the *Dmp1* null mice or that PTH has little effect on the defects in *Dmp1* null bone remodeling? Our future study seeks to understand the role of PTH in this animal model.

FGF-23 has been shown to be a potent phosphaturic hormone that promotes renal phosphate excretion,(11,12,29) and it is dramatically elevated in *Dmp1* null mice.(1) However, its pathogenic role has not been completely established owing to the fact that *Dmp1* null mice exhibit the phenotype of *Fgf23* null mice.(31) In this study, we applied neutralizing antibodies against FGF-23(20) into *Dmp1* null mice. We clearly showed that FGF-23 antibodies restored serum phosphate level and rescued the defects in the secondary ossification center in both long bone and vertebrae. The ex vivo metatarsal assay showed that the formation of secondary ossification was phosphate dose–dependent, and there was no difference between the control and *Dmp1* null metatarsals, suggesting that rescue of the secondary ossification center by FGF-23 antibodies likely was due to the restoration of phosphate homeostasis. However, this does not necessarily mean that the P_i_ is the only factor required for secondary ossification. It is known that FGF-23 targets primarily in the kidney to induce urinary phosphate excretion and suppress 1,25(OH)_2_D_3_ synthesis.(32,33) FGF-23 also acts directly on parathyroid glands to inhibit PTH synthesis and secretion.(34) Ironically, neither 1,25(OH)_2_D_3_ nor PTH seems to play an apparent role in the *Dmp1* null phenotype owing to the following two facts: (1) The serum level of 1,25(OH)_2_D_3_ is only slightly lower in *Dmp1* null mice, and (2) the PTH level in *Dmp1* null mice is 5 to 10 times higher than in the control mice.(1)

Interestingly, we noticed that in the long bone, the restoration of phosphate homeostasis by FGF-23 antibodies only partially rescued the bone-formation rate and osteomalacia, suggesting that DMP1 also plays a direct role in mineralization.

**Fig. 8:**
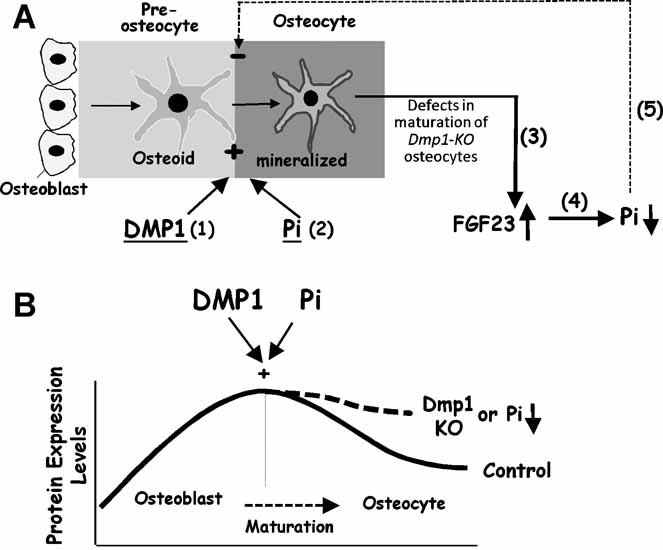
Model for effects of DMP1 and P_i_ on osteocyte differentiation and FGF-23 expression. (*A*), DMP1 (1) and P_i_ (2) are required for mineralization and maturation of osteoblasts into osteocytes; loss of DMP1 will lead to a sharp increase of FGF-23 in *Dmp1* KO osteocytes (3). An increase in FGF-23 results in hypophosphatemia (4). A reduction in P_i_ results in a decrease in mineralization, which, in turn, results in an impairment in osteoblast mineralization and differentiation (5). (*B*) Protein expression levels are high in matrix-producing osteoblasts, whereas protein expression levels are reduced during osteocyte maturation. DMP1 and P_i_ are two key players during this process. A loss of DMP1 or hypophosphatemia will disrupt normal osteoblast-to-osteocyte maturation, and expression of many genes, such as *osterix*, *Alp*, *Col1*, and *osteocalcin* remains abnormally elevated in embedded osteocytes.

It is well documented that there are dramatic downsizes in protein expressions and metabolic activity during the maturation of osteoblasts into osteocytes. This process is rather complex and largely unknown owing to technique difficulties that mature osteocytes reside in the mineralized matrix and that it is hard to isolate and study them in an in vitro condition created similar to their in vivo environment. However, our current studies suggest that DMP1 and phosphate homeostasis are likely the key factors in control of this downsizing process because there were abnormal increases in many osteoblast-specific proteins in the *Dmp1* KO osteocytes that are restored when phosphate homeostasis is normalized by neutralizing antibodies against FGF-23. These findings were incorporated into a model, as shown in Fig. 8.

However, this model, which provides a powerful framework for future studies in osteocyte maturation and mineralization, has certain limitations. For example, markers for early versus more mature osteocytes are not well characterized, and the morphologic characteristics of early versus mature osteocytes are still largely unknown. Second, the critical role of P_i_ in mineralization should not be purely limited to P_i_ itself because several reports demonstrated the importance of the ratio of P_i_/PP_i_ (inorganic pyrophosphate) on mineralization.(35–37) These studies showed that PP_i_, produced by the nucleoside triphosphate pyrophosphohydrolase, inhibits the formation and growth of hydroxyapatite crystals in mineralization. Non-tissue-specific alkaline phosphatase (TNAP) and Enpp1 are two key regulators of the extracellular PP_i_ concentrations required for controlled bone mineralization of PP_i_.

In conclusion, our current findings suggest that osteocytes not only control their own differentiation and mineralization but also may couple osteoclast formation, growth plate development, and the formation of secondary ossification centers through maintaining phosphate homeostasis via tightly controlled FGF-23 expression. FGF-23 antibodies may be developed for clinical treatment of hypophosphatemic disorders in the future.
